# Supplementary material for: Knocking Out OsAAP11 to Improve Rice Grain Quality Using CRISPR/Cas9 System
Source: Int J Mol Sci. 2023 Sep 21;24(18):14360. doi: 10.3390/ijms241814360 (PMC10532004; doi:10.3390/ijms241814360)
Supplement: Supplementary file 1 [file ijms-24-14360-s001.zip › ijms-2607364-supplementary.pdf]

Table S1. The primers used in this research.

Gene	Locus name	Forward Sequence (5′-3′)	Reverse Sequence (5′-3′)	Usage
Actin	*LOC_Os03g50885*	TGCTATGTACGTCGCCATCCAG	AATGAGTAACCACGCTCCGTCA	
OsAAP11	*LOC_Os11g09020*	GAAGGGGCACAAGAACCCGT	AGGCCAATCGTCGAATAGGTG	
OsGluA1	*LOC_Os01g55690*	CATTTGAGCCAATTCGGAGT	GGCCTGATTGTTGGAACTGT	
OsGluA2	*LOC_Os10g26060*	TGCTTGTTCCTCTTGTGCGA	TATGGCAACAACCGGCACTT	
OsGluA3	*LOC_Os03g31360*	GATTCGCACTGTAAGGTCCC	TGGCTTTGGCCTTCCAATTG	
OsGluB1a	*LOC_Os02g15169*	GCCAAAGTCAGAGCCAAAAG	GAACCAATGTGCAACACCAG	
OsGluB1b	*LOC_Os02g15178*	GCCAAAGTCAGAGCCAAAAG	GAACCAATGTGCAACACCAG	
OsGluB2	*LOC_Os02g15150*	CAATGATGGTGATGCATCGG	CTTGGTATTGAGCTTGTGC	
OsGluB4	*LOC_Os02g16830*	AGGCGTCAATGCATTGGTAG	GAGCTTGTGCTTGTTCCTGC	
OsGluB5	*LOC_Os02g16820*	AGGCGTCAATGCATTGGTAG	GAGCTTGTGCTTGTTCCTGC	
OsGluB6	*LOC_Os02g15070*	TGGAAGGATCACAAGTCTTG	GCATGGAATACTGAGTCTAC	
OsGluB7	*LOC_Os02g14600*	CTGGTGTACTGGGATTGACC	TGTTGATTGAGGGGTTGGAC	
OsGluC	*LOC_Os02g25640*	CACAAGGGCCAATAGCCAGA	GGTCACGTACATCACCGTGT	
OsGluD	*LOC_Os02g15090*	GGTGTCTTTGTCATTCGGCG	GAGCTTGGTCGCTCTGTCTT	
SSA2	*LOC_Os03g55730*	TGACGGTGTTCCACTTCCTC	GTCATACTCTGACAGCGGGG	
SSA3	*LOC_Os03g55734*	CGCCGCTACTACAATCACCA	CGTAGCTATCTGTGCCCGTC	
SSA4	*LOC_Os03g55740*	ACAGTGCCATTGTGGTGCTA	TTTGTTGTTGCCCGAGTCCA	
SSA5	*LOC_Os11g33000*	GGTAGCTCCACAGCCATGTT	TCACAGTTGCACTGAGGCAT	
Glb1	*LOC_Os05g41970*	AGGTTCCAGCCGATGTTCC	CATGCTCTCCTCGTAGCTCCTC	
RM1	*LOC_Os07g10580*	TTGCATGCAACGCTTCTGCA	CAAAGTAGACAACACCGACC	
PROLM1	*LOC_Os05g26240*	GGTAGCTCCACAGCCATGTT	TCACAGTTGCACTGAGGCAT	
PROLM4	*LOC_Os05g26350*	TCTAGTGTCCCGCAGCAATG	TGTTGCTGTAGCAGGACAGG	
PROLM10	*LOC_Os05g26460*	AAGTCTGGCAACAGCTCGC	AAGGGTGGTAATGGTACTGGGTG	
PROLM13	*LOC_Os05g26490*	AAGTCTGGCAACAGCTCGC	GCTTGAGCCAGATTCCGATCAAA	
PROLM14	*LOC_Os05g26620*	CTGCGCAGTTTGATGTTTTG	CTGGAGTTGTAGCTGCTGCA	
PROLM15	*LOC_Os05g26690*	CCTCTGCGCAGTTTGATGTT	GAGAGGCGACTGCAGCTAAT	qPCR
PROLM16	*LOC_Os05g26720*	CCCTTATTGCAATCAGCTGCA	AGCTTGAGCCAGATTCCGAC	
PROLM17	*LOC_Os05g26750*	TAGCATTACCACCCTTGGCG	TGGACATCCTTTATACGCTCCA	
PROLM18	*LOC_Os05g26770*	GCAATCAGCTGCGTTTCAGC	TGAGCTTGAGCTTGACCCAG	
PROLM19	*LOC_Os07g11900*	GGACATTAACGTTGTCCAGGC	GAGCCAGATTCCGGTCAATGTAGA	
PROLM20	*LOC_Os07g11920*	GGCAGCAGTATAGCATTGCG	CACCGCCAAGGGTGGTAATA	
PROLM23	*LOC_Os06g31060*	CGGCAAAACCCACAACCATT	GCTCAACACCCGTCACTGAT	
PROLM24	*LOC_Os06g31070*	TTGCTACTGGTCATTGCGGT	CCCACAATGCCTGTTTGCTT	
PROLM25	*LOC_Os07g10570*	CACTGTTGGTGGTGTCTGGT	AATTGTAAAGTCCCTGTGATGGT	
PROLM27	*LOC_Os12g16880*	CAGTGCAGCACAGTGGAAATC	AGAGCTTGAGTCTGATCGAAGT	
PROLM28	*LOC_Os12g16890*	CTTCCAATCACCCGTGTTTCAAC	CGCTAGCAAACTGTTGTAGCC	
PROLM29	*LOC_Os12g17010*	CAAGTTGAAACATCGGGGCG	ACCAAATGTAGGAACACATCGT	
PROLM30	*LOC_Os12g17030*	CGGTGAGTTCATAAGGCAGT	AGCTGTTGGCAGCACTGCTA	
AGPS2B	*LOC_Os08g25734*	AACAATCGAAGCGCGAGAAA	GCCTGTAGTTGGCACCCAGA	
AGPL2	*LOC_Os01g44220*	CTGAGGAAGAGGTGCTTTGG	TCTTTCGGGAGGATTGTGTC	
Wx	*LOC_Os06g04200*	TCCGAGAGGTTCAGGTCATC	ATGAGCTCCTCGGCGTAGTA	
SSI	*LOC_Os06g06560*	GGCAGTTGATATCAGTGACATATA	AAACTATATGTCACTGATATCAAC	

SSIIa	*LOC_Os06g12450*	GATCGACCAGGATGACGATT	GGGTAAAGCACCTGCAACAT	
SSIIIa	*LOC_Os08g09230*	GCCTGCCCTGGACTACATTG	GCAAACATATGTACACGGTTCTGG	
SSIIIb	*LOC_Os04g53310*	ATTCCGCTCGCAAGAACTGA	CAACCGCAGGATAACGGAAA	
SSIVa	*LOC_Os01g52250*	GGGAGCGGCTCAAACATAAA	CCGTGCACTGACTGCAAAAT	
BEI	*LOC_Os06g51084*	GGCATTGCACTCCAAAAGAT	GCTCCAGTTGTTGCCTTCTC	
BEIIa	*LOC_Os04g33460*	GCCAATGCCAGGAAGATGA	GCGCAACATAGGATGGGTTT	
BEIIb	*LOC_Os02g32660*	ATGCTAGAGTTTGACCGC	AGTGTGATGGATCCTGCC	
DBE1	*LOC_Os08g40930*	TGCTCAGCTACTCCTCCATCATC	AGGACCGCACAACTTCAACATA	
DBE2	*LOC_Os05g32710*	TAGAGGTCCTCTTGGAGG	AATCAGCTTCTGAGTCACCG	
Os07g0185300	*LOC_Os07g08770*	TTGGGGAACCACGAGAAGAG	GCTGTACACTGTACTGCACC	Off-target
Os01g0276700	*LOC_Os01g16960*	TGGGATCCGTGGGAGAAGTT	ACCTTGGTTGTATCCACCGC	sequencing
Hyg		GCTGTTATGCGGCCATTGTC	GACGTCTGTCGAGAAGTTTC	Transgenic component
cas9		ACCAGACACGAGACGACTAA	ATCGGTGCGGGCCTCTTC	detection
